# Predicting Solvation Free Energies of Molecules and Ions via First-Principles and Machine-Learning Molecular Dynamics

**DOI:** 10.1021/acs.jctc.6c00692

**Published:** 2026-06-29

**Authors:** Junting Yu, Shuo-Hui Li, Ding Pan

**Affiliations:** † Department of Physics, 58207 Hong Kong University of Science and Technology, Hong Kong, China; ‡ Department of Chemistry, 58207 Hong Kong University of Science and Technology, Hong Kong, China; § IAS Center for AI for Scientific Discoveries, 58207 Hong Kong University of Science and Technology, Hong Kong, China

## Abstract

The solvation free energy (SFE) of molecules and ions is a fundamental property governing their solvation behavior and solubility. Molecular simulations offer a route to compute SFEs using alchemical free energy methods such as thermodynamic integration or free energy perturbation. However, these methods suffer from the infamous end-point singularity, which leads to numerical instability when atoms approach closely, a challenge that becomes particularly acute in ab initio and machine-learning molecular dynamics simulations. Here, we introduce the bubble method to calculate the SFEs of molecules and ions from first-principles. Our approach avoids the end-state problem in both ab initio and machine-learning molecular dynamics simulations and is applicable to molecules and ions of arbitrary shape. When calculating the SFEs of ions using periodic density functional theory, we incorporate corrections for the neutralizing background charge, spurious interactions between periodic images, and the vacuum-water interface potential. To validate our method, we successfully computed the SFEs of methane, methanol, water, piperazine, and sodium and potassium ions using classical, ab initio, and machine-learning molecular dynamics simulations. Importantly, our method requires no experimental input or empirical data. This makes it particularly well-suited for studying systems under extreme conditions, such as high-pressure–temperature environments or under nanoconfinement, situations where experimental investigations are challenging and classical force fields, typically parametrized under ambient conditions, may be unreliable.

## Graphical abstract

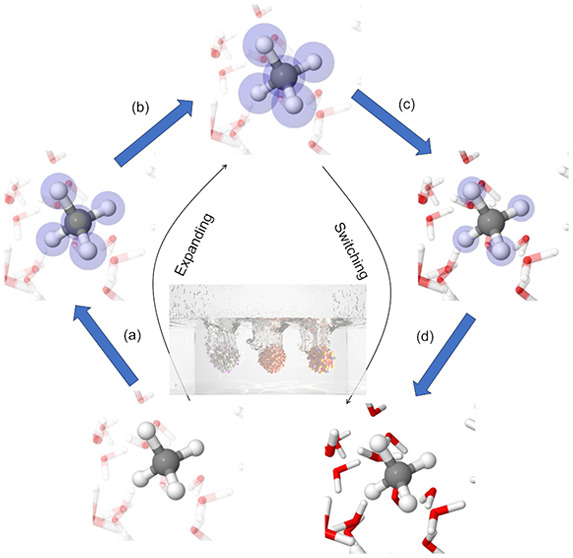

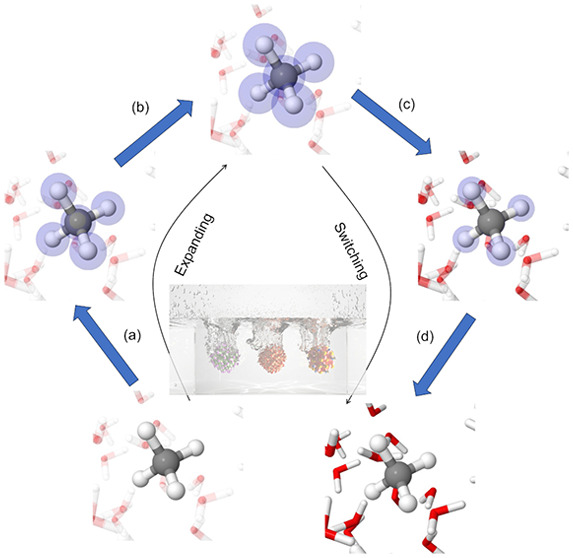

## Introduction

The maximum amount of a solute that can dissolve in a given solvent, defined as solubility, is a fundamental property driving processes in drug bioavailability, industrial extraction and purification, and geochemical systems. At the molecular level, this macroscopic phenomenon is governed by the solvation free energy (SFE), which quantifies the free energy change during the transfer of a solute from a vacuum into a solvent. In aqueous systems, this critical parameter is specifically termed the hydration free energy.^1–4^ Experimental measurements of SFEs are often challenging due to methodological uncertainties, particularly under elevated pressure (*P*) and temperature (*T*) conditions. Thus, accurate molecular-level calculations offer a very valuable alternative for determining these properties.

Implicit solvation models are widely used to compute solvation free energy (SFE) due to their efficiency.^5–7^ SFE is split into polar and nonpolar parts, typically modeled using the generalized Born (GB) approximation and solvent-accessible surface area, respectively.^8^ These methods may also integrate with electronic structure calculations like density functional theory (DFT).^9,10^ However, they rely on empirical parameters and ignore molecular solvent structure, limiting their accuracy under nonambient conditions such as extreme pressures, temperatures, or nanoconfinement.

With growing computational resources, explicit solvent models using molecular dynamics (MD) or Monte Carlo (MC) simulations are now widely used to compute solvation free energies (SFEs) via alchemical free energy methods, e.g., thermodynamic integration or free energy perturbation.^11–18^ Databases such as FreeSolv provide reference SFEs for neutral molecules from classical force fields and experiments.^3^ A key challenge for those methods is the end-point singularity: at full decoupling, Coulomb and van der Waals interactions diverge when atoms approach closely.^19^ This is addressed in classical force fields by soft-core potentials, which smooth interactions via an intermediate parameter,^19–21^ the dynamics in an unphysical “fourth dimension”,^22^ or serial atomic insertion.^23^ The soft-core potentials methods have been implemented in popular codes like GROMACS and LAMMPS.^24,25^

In first-principles DFT or machine-learning calculations, however, the implementation of analogous soft interactions remains an outstanding methodological challenge. In DFT calculations, electronic energies often fail to converge in self-consistent field methods when atoms are too close. Machine-learning potentials generally lack explicit physical forms for Coulomb and van der Waals interactions. Furthermore, their training data, derived from DFT or other high-level methods, rarely include configurations with extremely close atoms. As a result, machine learning potentials give unphysical energies when applied to such out-of-distribution geometries and thus need additional adjustments.^26–28^ An alternative method to solve the end-state problem is to construct a fictitious cavity around the solute molecule, thereby decomposing the alchemical free energy calculation into a series of subprocesses.^29–31^ This cavity method has been applied to compute SFEs of monatomic ions using first-principles MD simulations.^32^ However, for solute molecules or ions lacking spherical symmetry, this approach may exhibit numerical instability when employed in first-principles MD frameworks.

Here, we introduce the bubble method (Figure 1) to calculate the SFEs of molecules and ions from first-principles. Our method can avoid the end-point singularity in the alchemical free energy methods and is applicable to a wide range of molecular and ionic shapes and can be directly implemented in both ab initio and machine learning MD simulations. For calculating the SFEs of ions within periodic DFT, we incorporate corrections for the neutralizing background charge, spurious interactions between periodic images, and the vacuum-water interface potential. Importantly, our method does not rely on any experimental inputs or empirical data. This makes it particularly well-suited for studying systems under extreme conditions, such as high-pressure–temperature environments or under nanoconfinement, where experimental investigations are challenging and classical force fields, typically parametrized under ambient conditions, may be unreliable.

**Figure 1:**
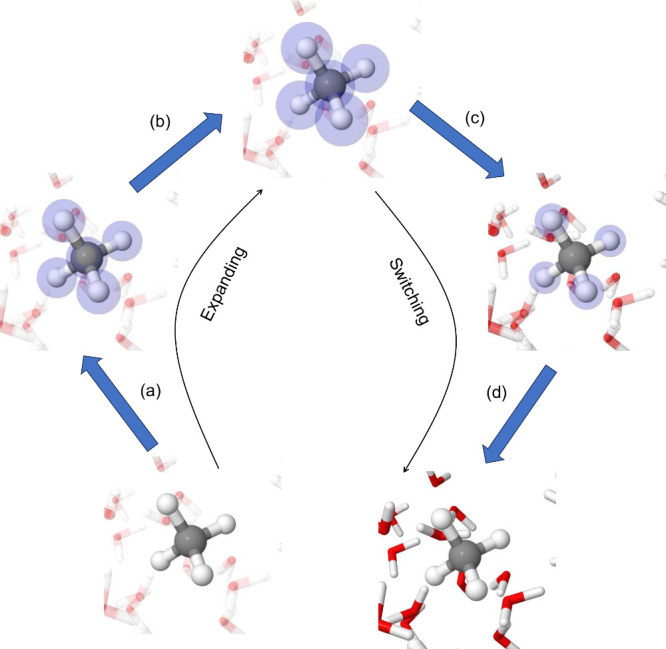
Schematic illustration of the bubble method scheme. (a) In the absence of solute–solvent interactions, a spherical bubble forms and expands around each atom in the solute molecule. (b) The bubbles reach their predefined maximum radius. (c) The bubbles begin to shrink, and the solute–solvent interactions are gradually switched on. (d) The bubbles have fully collapsed, and the system has returned to its fully interacting state.

## Results and Discussion

### Bubble Method

Here we apply the thermodynamic integration (TI) method to calculate the SFE (Figures 2 and 3), which creates a fictitious Hamiltonian *H*(λ) as a function of the coupling parameter λ. For the end state at λ = 0, the Hamiltonian *H*(0) = *H*_*m*_ + *H*_*w*_ represents the system where the solute molecule and solvent are noninteracting, while at λ = 1, the Hamiltonian *H*(1) = *H*_*mw*_ describes the fully solvated state, where the solute is fully dissolved and interacting with the solvent. Here we use the subscripts *m*, *w* and *mw* to denote the solute molecule, the solvent (e.g., water), and the solution system, respectively. The parameter λ, which runs from 0 to 1, represents a thermodynamic coupling process in which the interactions between the solute molecule and the solvent are gradually switched on. The free energy change for this process can be computed using the thermodynamic integration formula:

$$\Delta G=\int_{0}^{1}\left\langle \frac{\partial E\left(\lambda \right)}{\partial \lambda }\right\rangle d\lambda$$ 1

where the angle brackets ⟨⟩ denote an ensemble average, typically sampled via molecular dynamics or Monte Carlo simulations.

**Figure 2:**
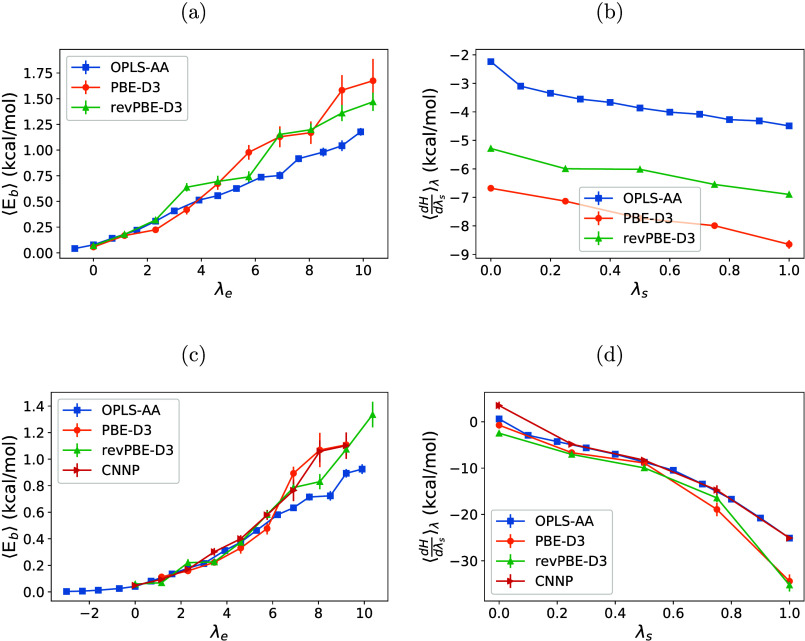
Integrands used in the thermodynamic integration to calculate the solvation free energy in the bubble method. (a) Expanding process of CH_4_(aq); (b) Switching process of CH_4_(aq); (c) Expanding process of H_2_O­(aq); (b) Switching process of H_2_O­(aq).

**Figure 3:**
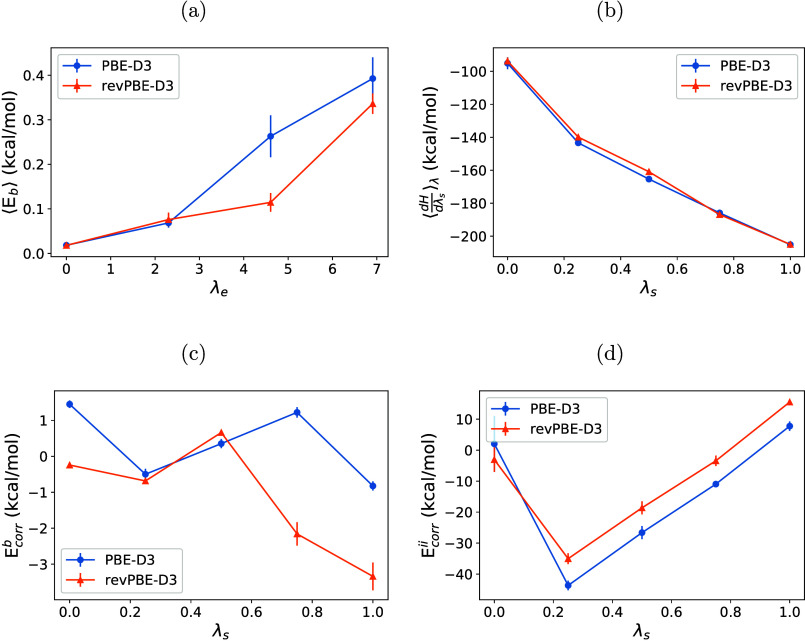
Integrands used in the thermodynamic integration and corrections to calculate the intrinsic solvation free energy of Na^+^(aq) in the bubble method: (a) expanding process, (b) switching process, (c) background correction, (d) ion–ion correction.

A common and straightforward choice for the Hamiltonian is the linear coupling form:

$$H\left(\lambda \right)=\left(1-\lambda \right)\left(H_{m}+H_{w}\right)+\lambda H_{mw}$$ 2

Substituting this into the integral yields

$$\Delta G=\int_{0}^{1}{\left\langle E_{mw}-E_{m}-E_{w}\right\rangle }_{\lambda }d\lambda$$ 3

where *E*_*x*_ represents the energy corresponding to the Hamiltonian *H*_*x*_.

However, this approach suffers from an end-state problem at λ = 0. In certain configurations, particularly when atoms of the solute and solvent closely approach or overlap, the energy difference *E*_*mw*_ – *E*_*m*_ – *E*_*w*_ can diverge. This singularity makes the integral in eq 3 challenging to evaluate numerically.^33^

To solve this issue, we introduce an artificial repulsive two-body bubble potential, defined as *U*_*b*_(*r*, λ_*e*_) = *u*(*r*)*e* ^λ_*e*_^. This potential acts on every atom pair between the solute molecule and the solvent. Its purpose is to create a “bubble expanding” effect that pushes solvent molecules away from the solute. The intermediate Hamiltonian for this process is then given by

$$H\left(\lambda_{e}\right)=H_{m}+H_{w}+\underset{i\in m,j\in w}{\sum }U_{b}\left(r_{ij},\lambda_{e}\right)$$ 4

where the parameter λ_*e*_ varies from –*∞* to a finite value λ_*E*_. The free energy change of this expanding process is

$$\Delta G_{e}=\int_{-\infty }^{\lambda_{E}}{\left\langle \underset{i\in m,j\in w}{\sum }U_{b}\left(r_{ij},\lambda_{e}\right)\right\rangle }_{\lambda_{e}}d\lambda_{e}$$ 5

where we have used the fact that the derivative of *U*_*b*_ with respect to λ_*e*_ is *U*_*b*_ itself. The two integral end points, –*∞* and λ_*E*_, correspond to 0 and 1, respectively, in eq 1. At this stage, there is no interaction between solute and solvent molecules. In numerical calculations, we can begin with a highly negative value of λ_*e*_, making the sum of *U*_*b*_ negligibly small. Then, we select a series of equally spaced λ_*e*_ values for separate simulations. This approach avoids the potential divergence of *H*_*mw*_.

Once the bubbles reach an appropriate size, we proceed to the next “switching” step. In this stage, we gradually turn on the interaction between the solute and solvent while simultaneously turning off all bubble potentials. We choose the simple linear form

$$H\left(\lambda_{s}\right)=\left(1-\lambda_{s}\right)\left(H_{m}+H_{w}+H_{b}\right)+\lambda_{s}H_{mw}$$ 6

where *H*_*b*_ is the sum of fully expanded bubble potentials in the *last* step of expanding:

$$H_{b}=\underset{i\in m,j\in w}{\sum }U_{b}\left(r_{ij},\lambda_{E}\right)$$ 7

Note that λ_*E*_ is fixed when varying λ_*s*_ in eq 6. The free energy change becomes

$$\Delta G_{s}=\int_{0}^{1}{\left\langle E_{mw}-E_{m}-E_{w}-E_{b}\right\rangle }_{\lambda_{s}}d\lambda_{s}$$ 8

If the bubbles expand sufficiently, the end-state problem at λ_*s*_ = 0 is avoided. It is suggested to choose a proper λ_*E*_ value so that the distance between solute and solvent molecules is similar to the average distance when the solute and solvent molecules interact physically. In numerical calculations, λ_*E*_ cannot be too large or too small: a value that is too large makes the numerical integral in the expansion process hard to converge, while a value that is too small leads to instability in the switching process (see the Supporting Information).

Alternatively, the switching step can be split into two separate steps: first, turning on the solute–solvent interaction, and then turning off the bubble potentials. The total free energy change from these two steps should be consistent with Δ*G*_*s*_, as free energy is a state function and independent of the path taken. However, this approach may introduce additional statistical uncertainties and increase computational cost.

The SFE can be calculated by the sum of the two steps,

$$\Delta G=\Delta G_{e}+\Delta G_{s}$$ 9

The remaining question lies on the form of *u*(*r*), which, in principle, does not affect the final result of SFE. An important requirement is that *u*(*r*) must be finite when *r* is small; otherwise the end-point singularity persists. In this study we mainly used the form *u*(*r*) = e^–*r*/*r*_0_^, where *r*_0_ = 0.2 Å^–1^. This is similar to the Buckingham potential, where the *r* ^–6^ term is set to 0. We also tested other parameter values and potential forms, such as the Gaussian form *u*(*r*) = e^–*Br* ^2^^, the exponential cubic form *u*(*r*) = e^–*Br* ^3^^ and the Fermi–Dirac potential *u*(*r*) = 1/(e^*B*(*r*–*r*_0_)^ + 1), in classical force field simulations. The details of these tests are shown in the Supporting Information. Our results show that the choice of parameters and potential forms does not significantly affect the final SFE. In the previous cavity method, the spherical cavity centered at the molecule was required to enclose the entire molecule.^29,30^ This poses challenges for numerical stability and statistical sampling when the molecule is nonspherical and the simulation box is small, as is often the case in AIMD simulations.

### Solvation Free Energies of Neutral Molecules

We selected three neutral molecules, methane, methanol, and water, to test our bubble method (Figure 2). While methane does not dissolve in water under ambient conditions, the other two do. Table 1 shows the calculated results by the classical OPLS-AA force field,^34^ and PBE-D3 ^35^ and revPBE-D3 ^36^ exchange-correlation functionals, in comparison with experimental values at *T* = 300 K and *P* = 10^5^ Pa.

**Table 1:** Solvation Free Energies of Neutral Molecules and Na^+^ Obtained by the Classical OPLS-AA Force Field, and PBE-D3 and revPBE-D3 Exchange Correlation Functionals^a^

molecule	OPLS-AA	PBE-D3	revPBE-D3	expt
CH_4_	2.28(0.08)	0.69(0.21)	1.92(0.13)	2.0
CH_4_ (sc)	12.45(0.08)	10.63(0.23)	–11.10(0.28)	
CH_3_OH	–4.29(0.15)	–10.51(0.31)	–6.33(0.28)	–5.1
H_2_O	–5.82(0.07)	–8.61(0.53)	–7.05(0.34)	–6.3
(CH_2_CH_2_NH)_2_	–6.95(0.12)	–13.64(0.53)	–10.17(0.54)	–7.4
Na^+^		–91.21(1.06)	–88.10(0.85)	–103.2∼−87.2
K^+^		–69.35(1.08)		–86.0∼−74.3

a Experimental values of the neutral molecules, except piperazine ((CH_2_CH_2_NH)_2_), are from ref 37 and piperazine from ref 38. The experimental value ranges of Na^+^ and K^+^ are from refs 39 and 32, respectively. Simulation uncertainties were estimated by using the blocking method^40^ and error propagation rules (unit: kcal/mol).

We found that the OPLS-AA force field yields the highest SFE, followed by revPBE-D3 and PBE-D3. Among these, OPLS-AA and revPBE-D3 show closer agreement with the experimental values. The nonpolar methane molecule has a positive SFE, meaning that it is more energetically favorable for methane to remain in the dilute gas phase than to dissolve in water. The polar water and methanol molecules containing an −OH group have negative SFEs and are easier to be dissolved into water. For the methane molecule, we also calculated its SFE at a supercritical condition (sc) at *T* = 1000 K and *P* = 1 GPa. Results of three methods are around 12 kcal/mol, which are 10 kcal/mol higher than those under ambient conditions.This indicates that it is less favorable for methane to move from the dilute gas phase into water at this high temperature than under ambient conditions. This does not mean, however, that methane is less soluble at this supercritical condition than at ambient conditions.

We further compared our method with the cavity method on piperazine ((CH_2_CH_2_NH)_2_), a six-membered heterocyclic organic compound. It is less spherical than the other tested molecules. The SFE obtained using our bubble method is −6.95 ± 0.12 kcal/mol, which agrees well with the experimental value of −7.4 kcal/mol. In contrast, the cavity method using the same artificial two-body potential, *u*(*r*)*e* ^λ_*e*_^, failed to yield a converged result when using 512 water molecules in a simulation box. When we turned on the real physical interactions between the solvent and solute, we need a large enough cavity, i.e. a large λ_*E*_, to maintain proper distances between solvent and solute molecules to avoid the dramatic change of the integral in the thermodynamic integration. However, a large λ_*E*_ causes the integrand to vary too much during the cavity expanding process, which again leads to numerical convergence problems. As a comparison, our method works with λ_*E*_ = 9.90, while the cavity method cannot obtain an integrable curve even with λ_*E*_ = 20.72 (see Figure S3­(c) in the Supporting Information). This test shows that our method is more numerically stable than the previous cavity method. The likely reason is that the atom pair interactions in the bubble method can delicately control the atomic distances between solute and solvent without seriously disrupting the solvation structure.

### H_2_O with the Neural Network Potential

Machine-learning techniques are now widely applied to construct potential energy surfaces using training data from first-principles calculations.^41,42^ This involves mapping atomic configurations into symmetry-invariant descriptors that are then used to predict the corresponding DFT-level energies and forces. This machine-learning approach achieves an accuracy comparable to first-principles methods while offering a significant improvement in computational efficiency.^43^ For example, Schran et al. trained a committee neural network potential (CNNP) model for water using the DFT data obtained from the revPBE0-D3 functional,^44^ which is one of the most accurate functionals for water systems.^45^

Here we demonstrate that our bubble method can be seamlessly integrated with a machine learning potential. As an example, we applied the pretrained Generation-1 CNNP model, which treats nuclei classically, to calculate the SFE of a water molecule. While the CNNP model accurately describes bulk water, it performs poorly for a single water molecule (see the Supporting Information). This is because the model was primarily trained and optimized on bulk water properties. Specifically, the average standard deviation across the 8 committee models for a single water molecule is as high as 5 kcal/mol, which could significantly impact the accuracy of the SFE calculation, so we directly calculated *E*_*m*_ using DFT with the revPBE0-D3 functional, and used the CNNP model to obtain *E*_*w*_ and *E*_*mw*_. The expanding and switching free energies were calculated as 4.588 ± 0.159 kcal/mol and −9.705 ± 0.357 kcal/mol, respectively, resulting in the SFE of −5.118 ± 0.391 kcal/mol. This value is slightly less negative than the experimental one (−6.3 kcal/mol).

If the neural network potential was trained including the monomer water data, then we do not need to calculate *E*_*m*_ using DFT. However, training such a neural network potential would be more difficult because potential descriptors are usually based on the local intermolecular environment of water molecules, which an isolated molecule lacks. A purely neural-network approach is not necessary. The main advantage of neural network potentials is that they can handle much larger systems accurately and efficiently, whereas calculating isolated molecules using DFT is computationally inexpensive.

### Solvation Free Energy of Charged Ions

The calculation of SFEs for ions presents greater challenges than those for neutral molecules, both experimentally and computationally. By definition, the absolute SFE of an ion corresponds to the free-energy change associated with transferring the ion from the gas phase into an infinitely dilute solution. However, neither reference state is experimentally accessible as an equilibrium system: isolated ions in the gas phase tend to associate with counterions, whereas ions in solution inevitably exist at finite concentrations near charged interfaces. Consequently, absolute ionic SFEs cannot be measured directly but must instead be inferred through combinations of equilibrium measurements, spectroscopic data, and thermodynamic cycles.^46^

In periodic DFT simulations, additional complications arise. The presence of a net charge necessitates corrections for both the neutralizing background charge and spurious interactions between periodic images. Moreover, because the electrostatic potential differs between vacuum and bulk water, a charged ion crossing the vacuum–water interface experiences a potential step, contributing to the overall solvation free energy.^47^ As a result, the experimental SFE of an ion is typically decomposed into two components: a bulk contribution arising from ion–water interactions in the interior (the intrinsic SFE), and a surface contribution originating from the vacuum–water interfacial potential. A range of methodological approaches have been developed to compute ionic SFEs, spanning classical force fields to DFT-based methods, and from continuum solvation models to fully atomistic explicit solvent representations. These studies have incorporated various correction schemes to address the aforementioned challenges.^1,4,32,48–52^ It is more challenging to apply the corrections in periodic DFT calculations than in classical force fields.^46,53–56^ Unlike in many classical force field simulations, where a charged ion is treated as a point charge with a fixed value, DFT calculations cannot ignore charge transfer between ion and water. The charge on the ion is strongly influenced by the configuration of its surrounding water molecules and the polarizability of the ion itself.^57^

When calculating the SFE of a charged ion using our method, we cannot directly evaluate the energy difference as in eq 3, because the charged and neutral systems have different energy references in DFT calculations with periodic boundary conditions. This issue is analogous to the treatment of charged defect formation energies in solids.^58^ To address this, we introduce two correction terms:

$$E_{\mathrm{corr}}=E_{\mathrm{corr}}^{b}+E_{\mathrm{corr}}^{ii}$$ 10

where *E*_corr_^*b*^ corrects for the fictitious interaction between the ionic charge and the neutralizing background charge, and *E*_corr_^*ii*^ accounts for the electrostatic interaction between the ion and its periodic images.

Periodic DFT calculations introduce a uniform background charge to neutralize the net charge in the unit cell, which leads to the fictitious interaction between the net charge and the background charge in the electrostatic energy (referred as the Hartree energy here). The solvation Hartree energy difference in eq 3 is calculated as

$$\Delta E^{H}=E_{iw}^{H}-E_{i}^{H}-E_{w}^{H}$$ 11

where *E*_*iw*_^*H*^ is the total electrostatic energy of the ion in water (*iw*), *E*_*i*_^*H*^ is the ion energy (*i*), and *E*_*w*_^*H*^ is for pure water (*w*). Considering the background neutralizing charge, we can decompose those Hartree energies as

$$E_{iw}^{H}=E_{iw-iw}^{H}+2E_{iw-b}^{H}+E_{b-b}^{H}$$ 12

$$E_{i}^{H}=E_{i-i}^{H}+2E_{i-b}^{H}+E_{b-b}^{H}$$ 13

$$E_{w}^{H}=E_{w-w}^{H}$$ 14

where the subscript *b* refers to the background charge. In periodic DFT, each Hartree term can be calculated as the electrostatic energy between the charge density ρ_*x*_ and potential *V*_*y*_ generated by the charge density ρ_*y*_

$$E_{x-y}^{H}=\frac{1}{2}\int_{\Omega }\rho_{a}\left(r\right)V_{y}\left(r\right)dr=\frac{2\pi }{\Omega }\underset{G}{\sum }\frac{{\mathrm{\rho ̃}}_{x}\left(G\right){\mathrm{\rho ̃}}_{y}\left(G\right)}{G^{2}}$$ 15

where the integral is calculated in one unit cell with the volume of Ω, and *ρ̃*(**G**) is the Fourier transform of ρ­(**r**) in reciprocal space. Note that the charge density ρ­(**r**) contains both electronic and nuclear charges. It is obvious that *E*_*x*–*y*_^*H*^ = *E*_*y*–*x*_^*H*^. It is worth noting that although *E*_*iw*_^*H*^ and *E*_*i*_^*H*^ are finite, each single term on the right-hand side of eqs 12 and 13 diverges due to the nonzero net charge (*ρ̃*_*x*_(**G** = 0) ≠ 0 for *x* ∈ {*iw*, *i*, *b*}).

After substituting eqs 12, 13, and 14 into eq 11, we obtain

$$\Delta E^{H}=\left(E_{iw-iw}^{H}-E_{i-i}^{H}-E_{w-w}^{H}\right)+2\left(E_{iw-b}^{H}-E_{i-b}^{H}\right)$$ 16

where the first term is the true electrostatic energy difference during solvation, and the second term accounts for the fictitious interactions with the background charge. Thus, the correction term *E*_corr_^*b*^ in eq 10 is

$$\begin{array}{rll} E_{\mathrm{corr}}^{b} & =-2\left(E_{iw-b}^{H}-E_{i-b}^{H}\right) & \\ & =-\int_{\Omega }\rho_{b}\left(r\right)\left[V_{iw}\left(r\right)-V_{i}\left(r\right)\right]dr=q\left({\mathrm{V̅}}_{iw}-{\mathrm{V̅}}_{i}\right) \end{array}$$ 17

where the background charge $\rho_{b}\left(r\right)=-\frac{q}{\Omega }$ , and *q* is the net charge of the ion in water. The potentials *V̅*_*iw*_ and *V̅*_*i*_ are the spatially averaged local potential in the ion–water and isolated ion systems, respectively, both of which are readily available in DFT calculations.

The other correction term *E*_corr_^*ii*^ comes from the electrostatic interaction between the ion and its periodic images. Experimental measurements are typically performed at low ion concentrations and subsequently extrapolated to the infinite dilution limit. In periodic DFT calculations, a low ion concentration requires a large unit cell, which is computationally expensive. The first term in eq 16 is

$$E_{iw-iw}^{H}-E_{i-i}^{H}-E_{w-w}^{H}=\frac{1}{2}\int_{\Omega }\left[\rho_{iw}\left(r\right)V_{iw}\left(r\right)-\rho_{w}\left(r\right)V_{w}\left(r\right)-\rho_{i}\left(r\right)V_{i}\left(r\right)\right]dr$$ 18

We consider the true electrostatic energy difference during solvation without ion–ion interactions as

$$E_{\mathrm{int}}^{H}=\frac{1}{2}\int_{\Omega }\left[\rho_{iw}\left(r\right)-\rho_{w}\left(r\right)\right]V_{w}\left(r\right)dr$$ 19

which represents the interaction energy between the charge density difference induced by solvation and the electrostatic potential of bulk water. Thus, the correction

$$E_{\mathrm{corr}}^{ii}=E_{\mathrm{int}}^{H}-\left(E_{iw-iw}^{H}-E_{i-i}^{H}-E_{w-w}^{H}\right)=\frac{1}{2}\int_{\Omega }\left[\rho_{i}\left(r\right)V_{i}\left(r\right)-\rho_{iw}\left(r\right)\delta V\left(r\right)\right]dr$$ 20

where δ*V*(**r**) = *V*_*iw*_(**r**) – *V*_*w*_(**r**).

The expanding, switching free energy values with the two corrections in eq 10 are listed in Table 2. Because there is only one ion in the unit cell, the bubble does not need to expand significantly, resulting in a small expansion free energy (Δ*G*_*e*_), as shown in Figure 3. Additionally, the background charge correction (*E*_corr_^*b*^) is also small, suggesting that the spatially averaged local potentials differ little between the isolated and dissolved ions. The switching contribution, accounting for ∼160 kcal/mol to the SFE, is the dominating part. The ion–ion correction term, *E*_corr_^*ii*^, further reduces the SFE by about 20 kcal/mol.

**Table 2:** Contributions to the Solvation Free Energy of Na^+^ ^a^

ion	functional	Δ*G*_*e*_	Δ*G*_*s*_	*E*_corr_^*b*^	*E*_corr_^*ii*^	*V*_s_(*V*)	Δ*G*_solv_
Na^+^	PBE+D3	1.22(0.09)	–162.35(0.53)	0.35(0.07)	–21.82(0.98)	3.963	–91.21(1.06)
Na^+^	revPBE+D3	0.82(0.05)	–160.667(0.40)	–0.11(0.01)	–14.88(0.91)	3.761	–88.10(0.85)
K^+^	PBE+D3	1.18(0.09)	–142.52(0.51)	0.24(0.07)	–19.64(0.95)	3.963	–69.35(1.08)

a All values are in kcal/mol, except for the surface potential (V).

### Surface Potential of Vacuum–Water Interface

In the experimental measurement of the ion SFE, the solvation process involves crossing the vacuum-water interface, and there is a potential jump at the water surface. This is also referred to as the mean inner potential of water in certain contexts.^59–61^ Thus, the experimental ion SFE contains the bulk and the surface contributions:

$$\Delta G_{\mathrm{ion}}=\Delta G_{\mathrm{bulk}}+\Delta G_{\mathrm{surf}}$$ 21

The surface contribution (Δ*G*_surf_) is proportional to the potential jump (*V*_s_) at the water surface, Δ*G*_surf_ = *qV*_s_. To calculate *V*_s_ from MD simulations, we built a water slab perpendicular to the *z* direction. We define an average electrostatic potential ϕ­(*z*) along *z*-axis as

$$\phi \left(z\right)=\frac{1}{A_{xy}}\left\langle \iint V\left(x,y,z\right)dxdy\right\rangle$$ 22

where *A*_*xy*_ is the area of the *xy*-plane, the double integral represents the spatial average over the *xy*-plane, ⟨·⟩ denotes the ensemble average. The electrostatic potential in the real space, *V*(*x*, *y*, *z*) can be obtained by the total charge density using Poisson’s equation or Green’s function

$$\nabla^{2}V\left(r\right)=-4\pi \rho_{t}\left(r\right)$$ 23

$$V\left(r\right)=\int \frac{\rho \left(r^{\prime }\right)}{\left|r-r^{\prime }\right|}dV^{\prime }$$ 24

We modifed the CP2K source code to calculate the spatial average ∬d*x*d*y*/*A*_*xy*_ on the fly during MD simulations.^62^

The ϕ­(*z*) of the water slab obtained using PBE-D3 and revPBE-D3 are shown in Figure 4. Given the symmetry of the curves about *z* = 25 Å, all data points are mapped to the left side for least-squares fitting. We used a logistic sigmoid (or hyperbolic tangent) function, as employed by Kathmann,^47^ to perform the fitting.

$$\mathrm{\phi ̂}\left(z\right)=\frac{V_{s}}{1+e^{-\beta \left(z-z_{0}\right)}}+V_{0}=V_{s}\mathrm{Sigm}\left[\beta \left(z-z_{0}\right)\right]+V_{0}=\frac{V_{s}}{2}\left(1+\tanh\left[\frac{\beta \left(z-z_{0}\right)}{2}\right]\right)+V_{0}$$ 25

where *V*_0_ is the reference potential, *z*_0_ denotes the position of the symmetry axis for ϕ­(*z*), and β controls the steepness of ϕ­(*z*) variation at the water surface. The fitted surface potentials *V*_*s*_ = 3.963 V (PBE-D3) and 3.761 V (revPBE-D3) align with previous computational and experimental values: 3.8 V (PBE),^60^ 3.63 V (PBE),^63^ 3.5 V (indirect calculation),^64^ and 3.5 V (electron holography).^59,65^

**Figure 4:**
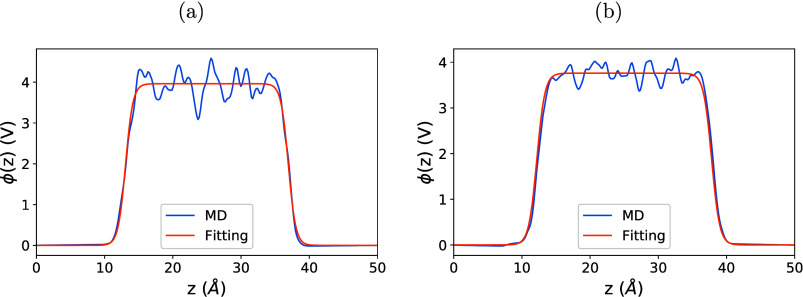
*z*-Dependence of the average electrostatic potential ϕ­(*z*) for water slabs, derived from AIMD simulations and subsequent model fitting. The system consists of 128 water molecules in a unit cell with dimensions 12.41 Å × 12.41 Å × 50 Å. The water molecules aggregate in the center of the cell, forming two distinct vacuum–water interfaces. Two exchange-correlation functionals are compared: (a) PBE-D3 and (b) revPBE-D3.

After considering the potential jump at the water surface, we obtained the SFE of −91.21 ± 1.06 kcal/mol for Na^+^ with the PBE-D3 functional and −88.10 ± 0.85 kcal/mol with revPBE-D3. These values are within the experimental range shown in Table 1. Recently, Lin et al. applied the cavity method to obtain the SFE of Na^+^ as 91.64 kcal/mol using the PBE functional and vdW corrections,^32^ which is very close to our value obtained by PBE-D3. In addtion, we tested our method on a second ion K^+^. Our SFE result is −69.35 ± 1.08 kcal/mol, which is again similar to the value (−65.81 kcal/mol) obtained by the cavity method in Lin et al’s study.^32^

## Conclusions

We introduce a numerically stable method for computing SFEs of neutral molecules and ions that can be readily integrated into both ab initio and machine learning MD simulations. This method avoids the end-point singularity in thermodynamic integration and works for any molecular or ionic shape. For ions treated within periodic density functional theory, we explicitly account for three key electrostatic corrections: the neutralizing background charge, finite-size artifacts arising from periodic image interactions, and the vacuum–liquid interface potential. We validated our method by calculating the SFEs of methane, methanol, water, piperazine, Na^+^, and K^+^ using classical, ab initio, and machine learning MD simulations. The accuracy of the resulting solvation free energies is determined only by the quality of the underlying potential energy surface (force field parameters or exchange–correlation functional), provided that MD simulations are well converged. Owing to its robustness and minimal reliance on system-specific empirical parameters, this method is especially advantageous for studying solvation under extreme thermodynamic conditions (e.g., high pressure and temperature) or in highly confined environments (e.g., nanopores, interfacial systems), situations where experimental data are scarce and conventionally parametrized classical force fields often become unreliable.

## Methods

### Classical Molecular Dynamics

We performed classical molecular dynamics simulations using the LAMMPS package.^25^ We used the OPLS-AA force field^34^ and the TIP3P water model.^66^ We used the shake algorithm to constrain their the bonds and angles of water molecules.^67^ Our unit cell contains one solute and 512 water molecules with periodic boundary conditions. In MD simulations we equilibrated each system in isobaric–isothermal (NPT) ensemble for 100 ps, followed by another ∼100 ps NPT production run. The time step is 2 fs, the temperature was controlled at 298 K using the Nose-Hoover thermostat,^68,69^ and pressure was set at 10^5^Pa with the Nose-Hoover barostat.^70^

#### Ab Initio Molecular Dynamics

We performed ab intio molecular dynamics (AIMD) simulations using the CP2K Quickstep package.^71,72^ We used the Goedecker–Teter–Hutter (GTH) norm-conserving pseudopotentials to describe core electrons^73,74^ and a triple-ζ doubly polarized (TZV2P) basis sets to expand the Kohn–Sham orbitals of valence electrons. The electron density cutoff is 340 Ry. We used deuterium instead of hydrogen to make use of a larger time step of 0.5 fs. There are one solute and 64 water molecules in a cubic unit cell with periodic boundary conditions. We applied the PBE^35^ and revPBE^36^ exchange-correlation functionals with the D3 van der Waals (vdW) correction.^75^ We began each AIMD trajectory with an initial NPT simulation of 10 ps. Temperature was regulated using the Bussi-Donadio-Parrinello thermostat (τ = 100 fs),^76^ and pressure was controlled via modified Hoover’s equations.^77^ We calculated the average cell lengths from the final 5 ps of this trajectory and then performed an NVT simulation for 20 ps using the resulting unit cell dimensions and the same thermostat to compute ensemble averages.

### Committee Neural Network Potential

For the water SFE calculations with the committee neural network potential (CNNP) model, we conducted the revPBE0-D3 calculations on both isolated molecules and bulk water (sampled every 100 MD steps) to compare energy differences between the hybrid functional and neural network potential, as shown in Table S5 in the Supporting Information. The bulk water has 64 molecules in the supercell with periodic boudary conditions. Our simulation parameters are the same as in the original CNNP paper:^44^ 400 Ry plane-wave cutoff, cpFIT3 auxiliary basis set for ADMM,^78^ and 6 Å Coulomb truncation for the Hartree–Fock exchange.

## Supplementary Material



## Data Availability

Input files and scripts are provided in the repository: https://github.com/JuntingYu1996/FPSFE.

## Notes

Solvation free energy (SFE) of methane in different forms of bubble potential using classical force field methods; Validation of the neural network potential energy for water; Numerical stability in thermodynamic integration (PDF)
