# Supplementary material for: Predicting Solvation Free Energies of Molecules and Ions via First-Principles and Machine-Learning Molecular Dynamics
Source: J Chem Theory Comput. 2026 Jun 29;22(13):6377–86. doi: 10.1021/acs.jctc.6c00692 (PMC13374039; doi:10.1021/acs.jctc.6c00692)
Supplement: Supplementary file 1 [file ct6c00692_si_001.pdf]

Supporting Information: Predicting Solvation Free Energies of Molecules and Ions via First-Principles and Machine-Learning Molecular Dynamics

Junting Yu,[1,*] Shuo-Hui Li,[1,†] and Ding Pan[1,2,3,‡]

[1]*Department of Physics, Hong Kong University of Science and Technology, Hong Kong, China*

[2]*Department of Chemistry, Hong Kong University of Science and Technology, Hong Kong, China*

[3]*IAS Center for AI for Scientific Discoveries, Hong Kong University of Science and Technology, Hong Kong, China*

(Dated: June 28, 2026)

Solvation free energy (SFE) of methane in different forms of bubble potential using classical force field methods

In this section we present some preliminary tests about SFE calculation of methane using classical force field potential. The test results of different bubble potential with different parameters are listed in Table S1, S2, S3 and S4. In these tables, λ_E is the maximum λ in the bubble potential $U_b(r, \lambda_e) = u(r)e^{\lambda_e}$. ΔG_e is the free energy change in the bubble expanding process. ΔG_s is the free energy change in the switching process. ΔG_{s1} is the free energy change in the switching process where only solute-solvent interactions are switched on, and then ΔG_{s2} is the free energy change where the bubble potential is switched off. $\Delta G = \Delta G_e + \Delta G_s$ is the final SFE result. All of the ΔG values are close to each other, with acceptable errors that come from numerical integrals, and also agree well with experimental value 2.0 kcal/mol. Figure S1 shows the total bubble potential energies as functions of λ_e.

In the expanding process, we chose 5-20 λ_e states. The lambda step size is ln(10)/3 $\approx$ 0.768 in the classical MD simulations, ln(10)/2 $\approx$ 1.15 in the AIMD simuations with neutral molecules, and ln(10) $\approx$ 2.30 in the AIMD simulations with ions. In the switching stage, we selected 11 and 5 λ_s states for classical MD and AIMD, respectively, to evenly divide λ_s from 0 to 1. For classical MD, we performed 50,000 steps at each lambda point, corresponding to a total simulation time of 100 ps given a timestep of 2 fs. For AIMD, we performed 40,000 steps at each lambda point, totaling 20 ps with a timestep of 0.5 fs. The total computational cost for calculating one SFE value using AIMD simulations is about 200,000 CPU core hours. The TI integral was evaluated using Simpson's rule. The error bar for each ensemble average was estimated via the blocking method [1], and the uncertainty of the final TI result was calculated using error propagation, treating each ensemble average as an independent error source.

Figure S2 shows the comparison between ΔG_s and $\Delta G_{s1} + \Delta G_{s2}$ of all testing results, indicating that there is no substantial difference whether the switching step is further de-

composed into two sub-steps.

TABLE S1: Solvation free energies obtained by different r_0 values in the Buckingham potential $u(r) = \mathrm{e}^{-\frac{r}{r_0}}$

r_0 (Å)	λ_E	ΔG_e (kcal/mol)	ΔG_s (kcal/mol)	ΔG_{s1} (kcal/mol)	ΔG_{s2} (kcal/mol)	ΔG (kcal/mol)
0.1	20.030	5.109 ± 0.054	-2.931 ± 0.014	-2.579 ± 0.017	-0.318 ± 0.004	2.213 ± 0.056
0.14	15.425	6.599 ± 0.078	-4.442 ± 0.013	-3.258 ± 0.012	-1.189 ± 0.013	2.152 ± 0.081
0.2	9.903	5.987 ± 0.077	-3.768 ± 0.015	-2.904 ± 0.014	-0.857 ± 0.009	2.219 ± 0.078
0.33	6.908	9.943 ± 0.086	-7.979 ± 0.051	-2.987 ± 0.014	-4.937 ± 0.041	2.019 ± 0.096
0.5	3.912	11.259 ± 0.123	-9.091 ± 0.054	-2.854 ± 0.013	-6.183 ± 0.047	2.222 ± 0.133
0.7	2.303	13.937 ± 0.119	-11.850 ± 0.056	-2.558 ± 0.013	-9.432 ± 0.046	1.947 ± 0.128
1.0	1.609	25.640 ± 0.202	-23.422 ± 0.136	-1.604 ± 0.015	-21.441 ± 0.139	2.594 ± 0.246

TABLE S2: Solvation free energies obtained by different B values in the Gaussian potential $u(r) = \mathrm{e}^{-Br^2}$

B (Å^{-2})	λ_E	ΔG_e (kcal/mol)	ΔG_s (kcal/mol)	ΔG_{s1} (kcal/mol)	ΔG_{s2} (kcal/mol)	ΔG (kcal/mol)
0.5	2.303	9.643 ± 0.099	-7.461 ± 0.043	-3.050 ± 0.013	-4.185 ± 0.041	2.409 ± 0.108
0.7	2.996	7.378 ± 0.082	-5.368 ± 0.027	-3.221 ± 0.011	-2.216 ± 0.021	1.942 ± 0.085
1.0	4.605	7.128 ± 0.069	-4.794 ± 0.020	-3.229 ± 0.010	-1.531 ± 0.017	2.368 ± 0.072
1.5	6.215	5.855 ± 0.053	-3.536 ± 0.013	-2.929 ± 0.012	-0.601 ± 0.007	2.325 ± 0.054
2.0	8.517	5.481 ± 0.081	-3.376 ± 0.013	-2.873 ± 0.011	-0.483 ± 0.005	2.125 ± 0.081

Validation of the neural network potential energy for water

Before implementing the SFE calculation, we selected a supercell containing 64 water molecules to test the energies. For each water molecule, we computed the energy of the

TABLE S3: Solvation free energies obtained by different B values in the exponential cubic potential $u(r) = \mathrm{e}^{-Br^3}$

B (Å^{-3})	λ_E	ΔG_e (kcal/mol)	ΔG_s (kcal/mol)	ΔG_{s1} (kcal/mol)	ΔG_{s2} (kcal/mol)	ΔG (kcal/mol)
0.5	5.298	6.247 ± 0.055	-4.119 ± 0.017	-3.204 ± 0.011	-0.918 ± 0.008	2.125 ± 0.057
0.7	6.215	5.336 ± 0.049	-3.257 ± 0.013	-2.835 ± 0.013	-0.423 ± 0.005	2.078 ± 0.051
1.0	8.517	5.337 ± 0.077	-2.908 ± 0.014	-2.601 ± 0.016	-0.288 ± 0.004	2.449 ± 0.079
1.5	13.816	5.145 ± 0.063	-3.155 ± 0.024	-2.712 ± 0.015	-0.332 ± 0.006	2.101 ± 0.065

TABLE S4: Solvation free energies obtained by different B and r_0 values in the Fermi potential $u(r) = \frac{1}{\mathrm{e}^{B(r-r_0)}+1}$

B (Å^{-1})	r_0 (Å)	λ_E	ΔG_e (kcal/mol)	ΔG_s (kcal/mol)	ΔG_{s1} (kcal/mol)	ΔG_{s2} (kcal/mol)	ΔG (kcal/mol)
10	1.0	10.820	5.543 ± 0.043	-3.348 ± 0.012	-2.853 ± 0.012	-0.451 ± 0.005	2.2391 ± 0.045
10	1.5	6.215	5.737 ± 0.055	-3.556 ± 0.011	-2.986 ± 0.012	-0.567 ± 0.007	2.185 ± 0.057
10	2.0	2.303	6.205 ± 0.077	-4.120 ± 0.019	-3.200 ± 0.011	-0.918 ± 0.009	2.087 ± 0.078
20	2.0	2.303	5.450 ± 0.068	-3.096 ± 0.014	-2.767 ± 0.014	-0.344 ± 0.004	2.339 ± 0.070

isolated molecule as well as that of the remaining 63 molecules. After obtaining 64 such pairs of results, we evaluated the ensemble average. We also compared the committee neural network potential (CNNP) results [2] with revPBE0-D3 results; the data are reported in Table S5. As shown in Table S5, for the energies of 64 water molecules (E_{mw}) and 63 water molecules (E_w), the CNNP results are close to the revPBE0-D3 results, with a standard deviation among the eight committee models of only about 0.7 kcal/mol. However, for a single water molecule, the NNP overestimates the energy by approximately 8 kcal/mol, and the standard deviation is as large as ∼5 kcal/mol.

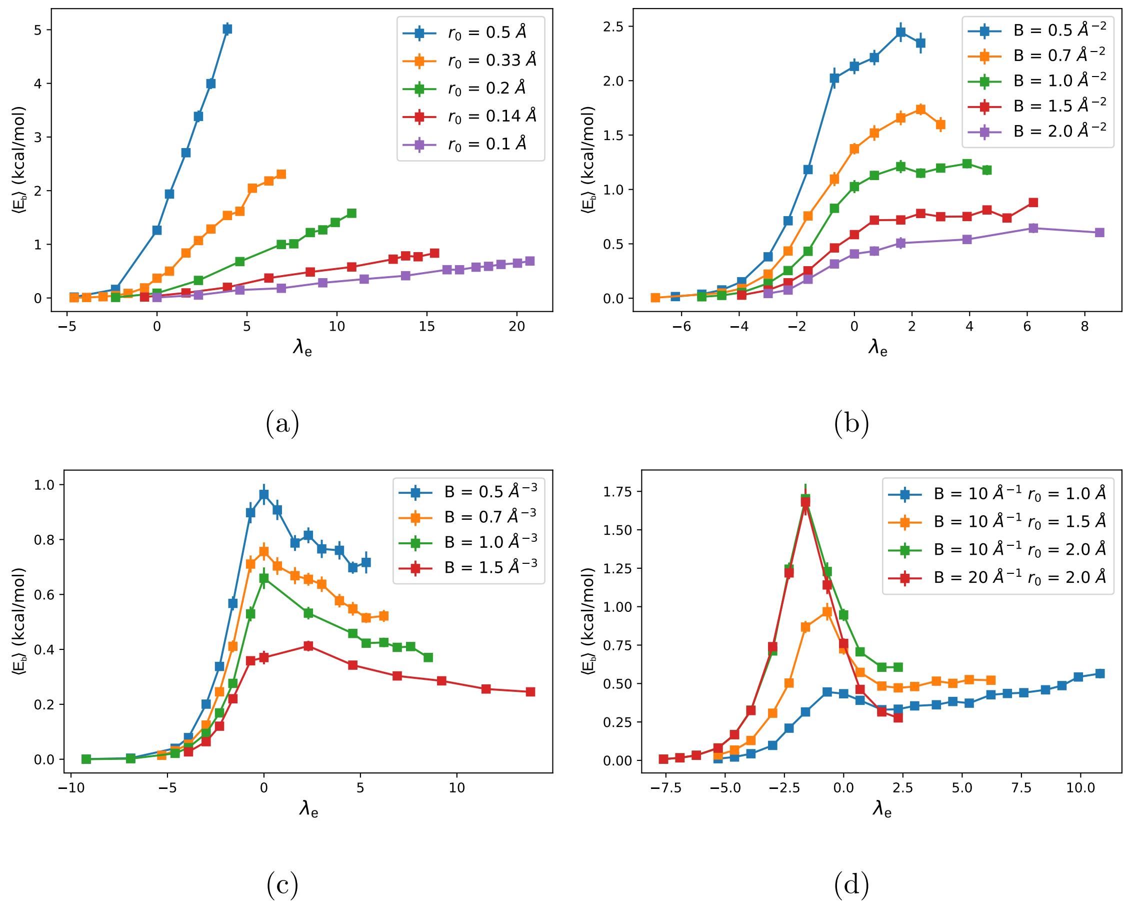

FIG. S1: Energies during the bubble forming by four bubble potentials. (a) $u(r) = \mathrm{e}^{-\frac{r}{r_0}}$. (b) $u(r) = \mathrm{e}^{-Br^2}$. (c) $u(r) = \mathrm{e}^{-Br^3}$. (d) $u(r) = \frac{1}{\mathrm{e}^{B(r-r_0)}+1}$.

TABLE S5: Energy validation of the committee neural network potential. The unit of energy is kcal/mol.

$\langle E_m^{dft} - E_m^{nnp} \rangle$	$\langle \sigma_m \rangle$	$\langle E_w^{dft} - E_w^{nnp} \rangle$	$\langle \sigma_w \rangle$	$\langle E_{mw}^{dft} - E_{mw}^{nnp} \rangle$	$\langle \sigma_{mw} \rangle$	$\langle \Delta E^{dft} - \Delta E^{nnp} \rangle$
-8.330	5.186	1.669	0.748	2.142	0.770	8.803

Numerical stability in thermodynamic integration

In our bubble method, an adequate bubble size removes the end-state divergence at $\lambda_s = 0$ in the switching process. As shown in Fig.S3(a) for CH_3OH, the green curve calculated with $\lambda_E = 9.90$ is a better choice than the blue curve calculated with $\lambda_E = 8.52$, because it is

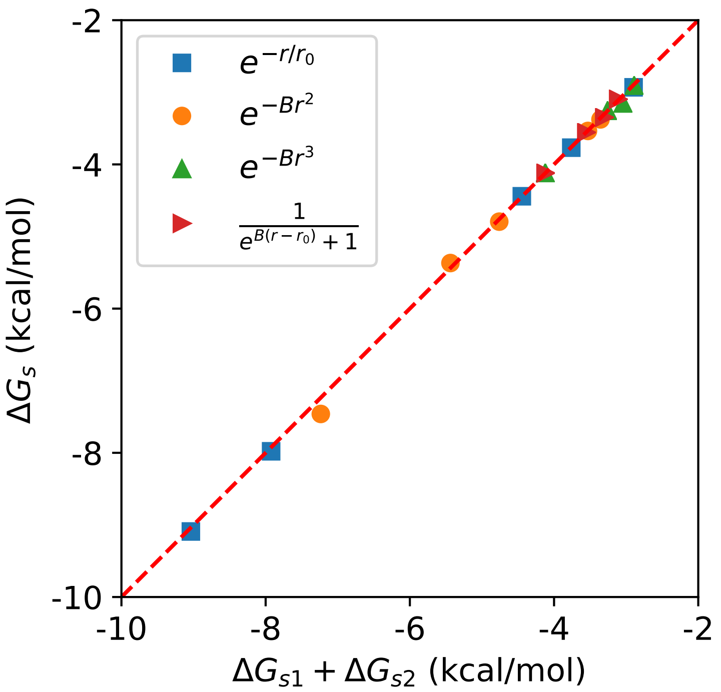

FIG. S2: Comparison between ΔG_s and $\Delta G_{s1} + \Delta G_{s2}$.

flatter and easier for numerical integration. Fig.S3(b) shows the inserting stage of CH_3OH using the cavity method. A larger $\lambda_E = 16.12$ is required for easy numerical integration, which can be a source of numerical instability in the cavity method. For a larger molecule like piperazine, shown in Fig.S3(c), $\lambda_E = 20.72$ is far from sufficient to produce an integrable curve in the inserting process.

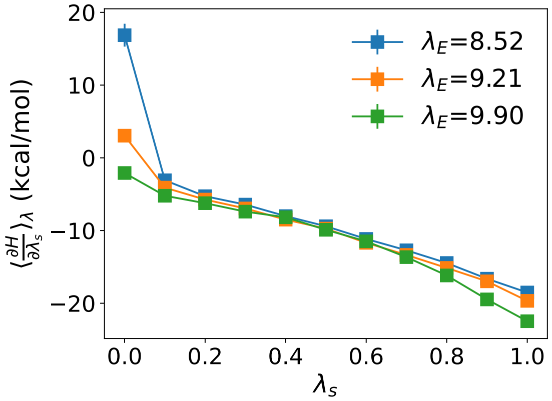

(a)

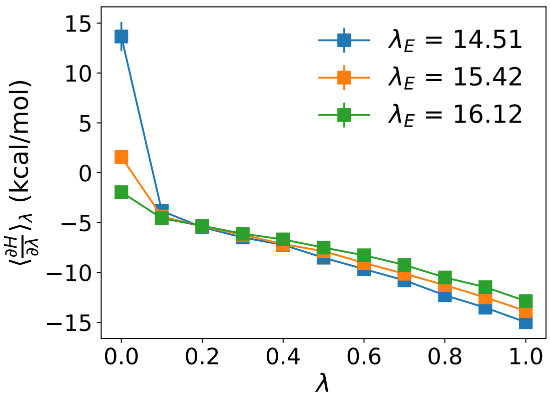

(b)

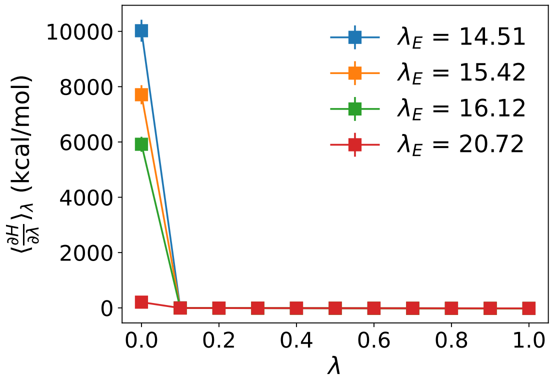

(c)

FIG. S3: Integrands as function of λ_s or λ in the thermodynamic integration in the bubble and cavity methods. (a) Switching process of CH_3OH in our bubble method. (b) Inserting process of CH_3OH in the cavity method (c) Inserting process of piperazine ($C_4H_{10}N_2$) in the cavity method. In the inserting process of the cavity method, the Hamiltonian $H(\lambda) = (1-\lambda)(H_m + H_w + H_c) + \lambda H_{mw}$, where the cavity potential $H_c = u(r)e^{\lambda_E}$. To ensure the stability of numerical integration, λ_E should be large enough to make the integrands change smoothly. However, λ_E can not be too large, as shown in (c), because the expanding process in the bubble or cavity method may be numerically unstable.

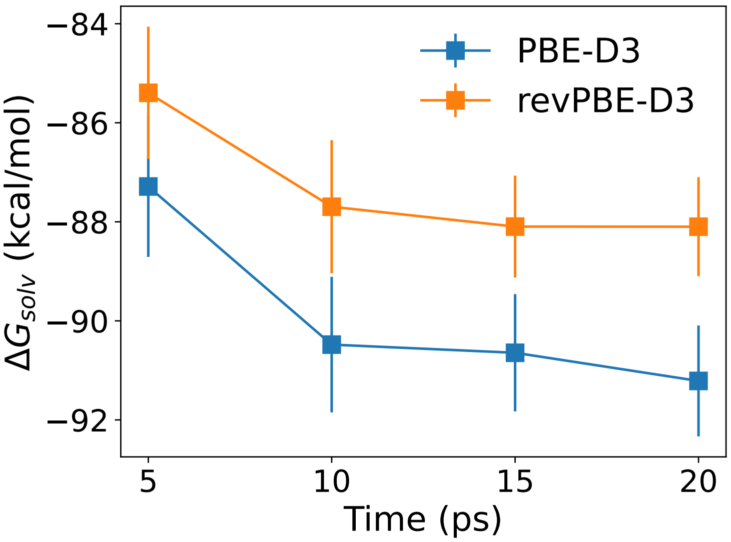

FIG. S4: Solvation free energy of Na^+ calculated using the bubble method as a function of AIMD simulation time.

* junting.yu@connect.ust.hk

† shuohuili@ust.hk

‡ dingpan@ust.hk

[1] Flyvbjerg, H. & Petersen, H. G. Error estimates on averages of correlated data. *The Journal of Chemical Physics* **91**, 461–466 (1989).

[2] Schran, C., Brezina, K. & Marsalek, O. Committee neural network potentials control generalization errors and enable active learning. *The Journal of Chemical Physics* **153**, 104105 (2020).
